# Staphylokinase has distinct modes of interaction with antimicrobial peptides, modulating its plasminogen-activation properties

**DOI:** 10.1038/srep31817

**Published:** 2016-08-24

**Authors:** Leonard T. Nguyen, Hans J. Vogel

**Affiliations:** 1 Biochemistry Research Group, Department of Biological Sciences, University of Calgary, Calgary, Alberta, Canada

## Abstract

Staphylokinase (Sak) is a plasminogen activator protein that is secreted by many *Staphylococcus aureus* strains. Sak also offers protection by binding and inhibiting specific antimicrobial peptides (AMPs). Here, we evaluate Sak as a more general interaction partner for AMPs. Studies with melittin, mCRAMP, tritrpticin and bovine lactoferricin indicate that the truncation of the first ten residues of Sak (SakΔN10), which occurs *in vivo* and uncovers important residues in a bulge region, improves its affinity for AMPs. Melittin and mCRAMP have a lower affinity for SakΔN10, and in docking studies, they bind to the N-terminal segment and bulge region of SakΔN10. By comparison, lactoferricin and tritrpticin form moderately high affinity 1:1 complexes with SakΔN10 and their cationic residues form several electrostatic interactions with the protein’s α-helix. Overall, our work identifies two distinct AMP binding surfaces on SakΔN10 whose occupation would lead to either inhibition or promotion of its plasminogen activating properties.

*Staphylococcus aureus* is a major pathogen that causes significant morbidity and mortality^1^. The rapid evolution of the organism has led to significant global challenges, from the emergence of methicillin-resistant *S. aureus* (MRSA) in the 1960’s to the rise of community-acquired MRSA in the 1990’s and its recent endemic appearance in hospital settings^2^,^3^. The divergence of *S. aureus* strains is mostly caused by the transfer of mobile genetic elements such as plasmids, transposons, and bacteriophages, some of which encode several virulence factors that increase pathogenicity^4^.

Staphylokinase (Sak) is one such factor that is secreted by most lysogenic *S. aureus* strains^5^,^6^,^7^. Although Sak plays a role in the establishment of infections in humans, high levels of the protein are also associated with a decrease in disease severity and mortality^6^,^8^. In the blood, Sak achieves its function primarily by forming a plasminogen activating complex together with plasmin itself, which prevents biofilm formation^9^ and more importantly, initiates the fibrinolytic cascade to help the invading bacterium move deeper into tissues^10^. Consequently, Sak has been intensively studied and it has been developed as a novel thrombolytic drug to treat acute myocardial infarctions and embolisms, for example^11^.

A secondary function of Sak is its ability to neutralize host antimicrobial peptides (AMPs). AMPs constitute a wide range of peptides that are produced by all mammals and other organisms as a defense mechanism against invading pathogens^12^,^13^,^14^. Sak was first found to be able to bind to human α-defensins, which are small β-sheet peptides that are mainly secreted by neutrophils and are prominent in human airways^15^. These human neutrophil proteins (HNPs) are also expressed at high levels in the intestine^16^. The formation of a complex between Sak and HNPs results in the mutual inhibition of the peptide and protein’s bactericidal and thrombolytic activities, respectively. Using antibody detection methods, Sak has been shown to bind to human LL-37 and mouse cathelicidin-related antimicrobial peptide (mCRAMP)^17^. In contrast to the effects seen for the α-defensins, the binding to these particular peptides seems to increase the plasminogen activation properties of Sak. Hence, *S. aureus* is thought to exploit these peptides, which are present in the early stages of pneumonia, to enhance Sak enzymatic activity and promote bacterial spreading^17^.

The secretion of staphylokinase is one of several mechanisms that *S. aureus* has developed to overcome the bacteriostatic effects of antimicrobial peptides^18^. Other evasion mechanisms utilized by *S. aureus* involve AMP sensing two-component systems^19^, covalent modifications of surface lipids to decrease their negative charge such as aminoacylation of phosphatidylglycerol^20^ and D-alanylation of teichoic acids^21^, as well as secretion of degrading proteases such as *S. aureus* V8 protease or aureolysin^22^. There are parallel mechanisms in other pathogenic bacterial species where secreted proteins sequester and inhibit host antimicrobial peptides and proteins. For example, the Ivy proteins are produced by several Gram-negative bacteria to inihibit the cell wall hydrolyzing activity of lysozyme^23^,^24^. As well, some *Streptococcus pyogenes* strains secrete streptococcal inhibitor of complement, a multifunctional 30.6 kDa protein that interferes with the human complement system and inhibits lysozyme, LL-37, and both human α- and β-defensins^25^,^26^,^27^.

In this study, we sought to investigate the potential of Sak to interact with different AMPs. AMPs are generally known for being cationic and amphipathic, however they have widely diverse secondary and tertiary structures and can employ distinct mechanisms of antimicrobial action^28^,^29^. There is great interest in AMPs as potential therapeutics to combat the problem of antibiotic resistance^30^,^31^. In addition to mCRAMP, we tested melittin as a classical peptide that adopts an α-helical amphipathic conformation upon membrane binding. Although it can act as an AMP, melittin is a cytotoxic peptide that is derived from honey bee venom^32^. Tritrpticin is a cathelicidin derived from porcine neutrophils that belongs to the Trp- and Arg-rich family of AMPs^33^. It adopts a unique two-turn structure in the presence of membranes^34^. Bovine lactoferricin (LfcinB) is another Trp- and Arg-rich peptide that is derived through intestinal proteolysis of the milk protein lactoferrin and it adopts an amphipathic β-hairpin structure in aqueous solution^35^,^36^. Together, these four AMPs represent amphipathic α-helical, β-hairpin and turn-like structures.

Staphylokinase contains 136 amino acids and has a molecular weight of 15.5 kDa. Its 3D structure features a central α-helix lying across a five-stranded β-sheet^37^,^38^. The N-terminal sequence of Sak is known to be subjected to truncation by proteolytic processing *in vivo*. While the full-length protein can associate with plasmin, hydrolysis at the Lys10-Lys11 peptide bond is required for the complex to become fully active^39^. Plasmin itself can act as the activating protease for Sak. However, the overexpression of the protein in *E. coli* and *B. subtilis* strains can result in the recovery of three forms of Sak with different N-termini: the full-length protein, one with a six-residue deletion (SakΔN6), and one with a ten-residue deletion (SakΔN10)^40^,^41^,^42^. When Sak was purified from a culture of an *S. aureus* strain that was selected for its high plasminogen activating activity, the purified protein showed a single observable band on SDS-PAGE, and this form was identified as SakΔN10^42^.

Here, we evaluate the specificity of two forms of staphylokinase (wtSak and SakΔN10) for their capacity to bind different AMPs using isothermal titration calorimetry (ITC) and nuclear magnetic resonance (NMR) spectroscopy. Our results indicate that SakΔN10 is the preferred partner for AMPs, and that these four AMPs target two different regions on the protein’s surface. The two distinct protein-peptide complexes may explain the different effects seen on the plasminogen activating properties of staphylokinase.

## Results

### ITC: selectivity and thermodynamics of Sak-AMP interactions

In order to determine the interaction strength of different antimicrobial peptide-Sak pairings, a series of ITC experiments were performed (Table 1). LfcinB and Tritrp are moderate-affinity binding partners for Sak, with respective dissociation constants of 27.2 μM and 11.2 μM. Their isotherms show that they interact with Sak at 1:1 ratios and with exothermic heats of binding. The cathelicidin mCRAMP shows no binding in our tested conditions. This result is unexpected because mCRAMP has previously been found to associate with Sak using polyclonal antibody detection methods^17^. mCRAMP has a net +6 charge at neutral pH compared to +7 and +4 for LfcinB and Tritrp, respectively, therefore there should be a similar potential for electrostatic interactions between these AMPs and a negatively charged surface on Sak. Due to the temperature dependence on binding enthalpy^43^, it is possible that there is a high affinity between the two molecules and that the titration with mCRAMP may have been set at a temperature where enthalpy is minimal. For this reason, we repeated the experiment at 37 °C. This result, as well as the titration data from our NMR experiments, confirms the lack of strong binding (K_D_ < 10^−4^ M) between mCRAMP and full-length Sak (not shown). In the titration of melittin to Sak, the thermogram shows very small endothermic heats of interaction which presented difficulties in curve fitting and determining an accurate dissociation constant.

**Figure 1:**
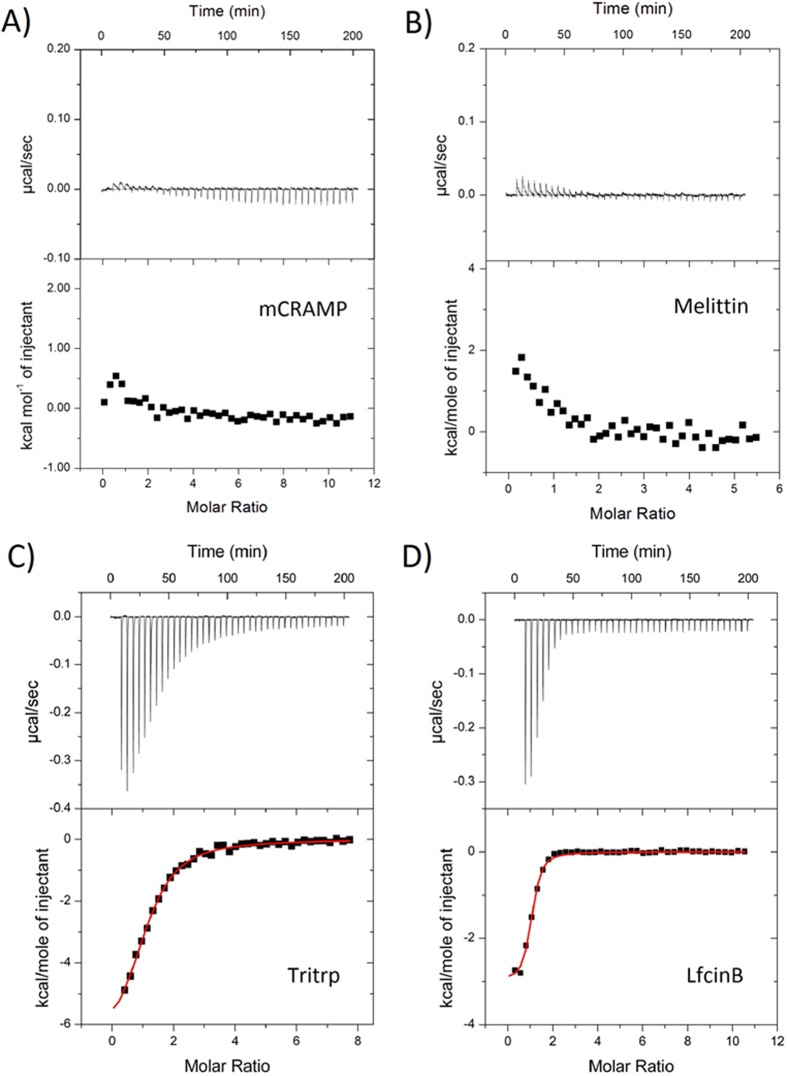
ITC isotherms obtained for peptide injections into a solution of SakΔN10 performed at 25 °C. (**A**) mCRAMP, (**B**) melittin, (**C**) Tritrp, and (**D**) LfcinB. For LfcinB and Tritrp, the integrated heats were fitted using the one site binding model. For mCRAMP and melittin, the weak binding profiles make reliable curve fitting difficult. See Table 1 for thermodynamic parameters.

In a second set of ITC experiments, we used SakΔN10, an *in vivo* Sak variant with its first ten residues truncated. Overall, SakΔN10 was found to be a better binding partner for all AMPs tested (Fig. 1). The dissociation constants for LfcinB and Tritrp are in the low micromolar range. In particular, LfcinB has a *K*_*D*_ of 0.37 μM, which is two orders of magnitude lower compared to full length Sak. The titration with mCRAMP now shows small endothermic heats approaching the profile observed with melittin. This suggests that the weak association between SakΔN10 and these peptides may be driven in part by hydrophobic interactions. The curves become mostly flat after 2:1 peptide:SakΔN10, therefore the stoichiometry value *N* for mCRAMP and melittin is likely to be 1, in agreement with the stoichiometry observed for LfcinB and Tritrp.

### NMR assignments and structural analysis of Sak and SakΔN10

^1^H,^15^N HSQC NMR spectra were acquired to monitor the structural changes of ^15^N-labeled Sak or SakΔN10 upon the addition of AMPs. Main chain residue assignments were made using three-dimensional backbone experiments on ^13^C,^15^N-labeled protein samples, and they were verified against a previously published NMR assignment of staphylokinase (Supplementary Fig. 1)^37^. Most of the non-proline residues could be assigned except for the first few N-terminal residues and the following residues: Lys57, Lys74, and Ile108. The peaks for these residues have likely undergone broadening due to chemical exchange. While the amide peak for Tyr44 is not present in the Sak spectrum presumably due to chemical exchange with the solvent^37^, it appears in the SakΔN10 spectrum. For both forms of the protein, the histidines of the C-terminal His_6_-tag were left unassigned. The positions of the vast majority of the amide peaks remain largely unchanged between Sak and SakΔN10, which indicates that the truncation of the first ten residues does not affect the overall fold of the protein.

**Figure 2:**
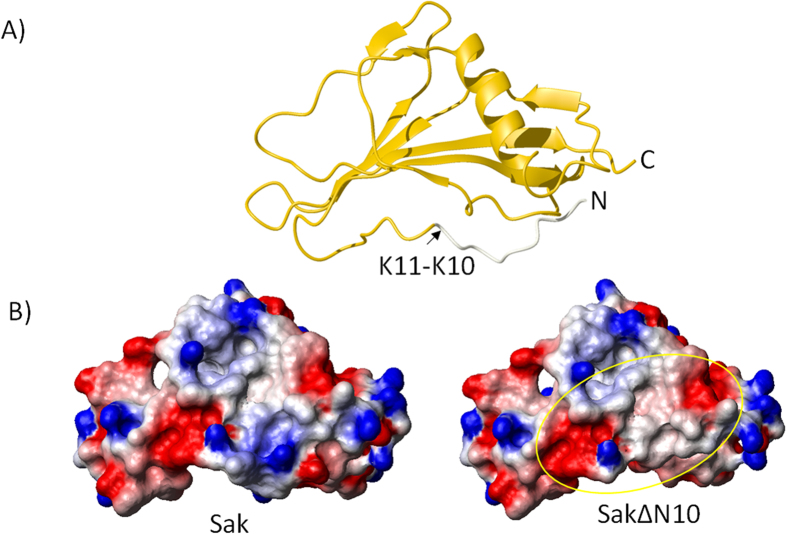
(**A**) Topology of Sak. Source: PDB ID 1SSN^37^. (**B**) Electrostatic surface potential of Sak (left) and SakΔN10 (right), both viewed at the same angle as in Panel A. The large negative surface of SakΔN10 is highlighted.

The topology of staphylokinase consists of a central α-helix (Lys57-Asp69) adjacent to a short two-stranded β-sheet, all of which lie perpendicular on a five-stranded β-sheet (Fig. 2A)^37^,^38^. The N-terminal segment, which encompasses the first ten residues and extends to Glu19, lacks regular secondary structure and folds back to make several contacts to different parts of the protein. However, heteronuclear ^15^N-{^1^H} NOE data indicated that this region has higher conformational variability than the remainder of the protein^37^. In the X-ray crystal structure of Sak, residues 1–15 are in fact missing due to low electron density observed^38^. Using the mean NMR solution structure as a starting point (PDB 1SSN)^37^, residues 1–10 (SSSFDKGKYK) were removed to create a model structure for SakΔN10 and evaluate its electrostatic surface potential (Fig. 2B). With its missing electron density for the N-terminal residues, the X-ray crystal structure also presents a potential model for SakΔN10, however there are subtle differences between the structures due to the different experimental approaches (NMR vs. X-ray), which would make it difficult to make a direct comparison. Overall, there are several negatively charged surfaces on Sak interrupted by scattered positive charges. The N-terminal deletion of residues 1–10 lowers the pI of SakΔN10 from 6.15 to 5.47 and it removes a small cluster of positively charged residues. Lys11 is likely to be very mobile as it is now the new N-terminus of the SakΔN10 protein. The solvent exposure for all residues of Sak and SakΔN10 were calculated by MOLMOL^44^ and plotted in Fig. 2C. The biggest increases in exposure are found for the new N-terminus Lys11 (38.4% to 65.8%), His43 (29.7% to 39.5%), Tyr44 (25.2% to 39.2%), Ala72 (13.0% to 33.1%), and Glu75 (20.4% to 28.9%). The newly exposed areas are mostly uncharged, which creates a larger uninterrupted anionic surface on the protein.

### NMR titration experiments: mapping a surface for AMP interaction

**Figure 3:**
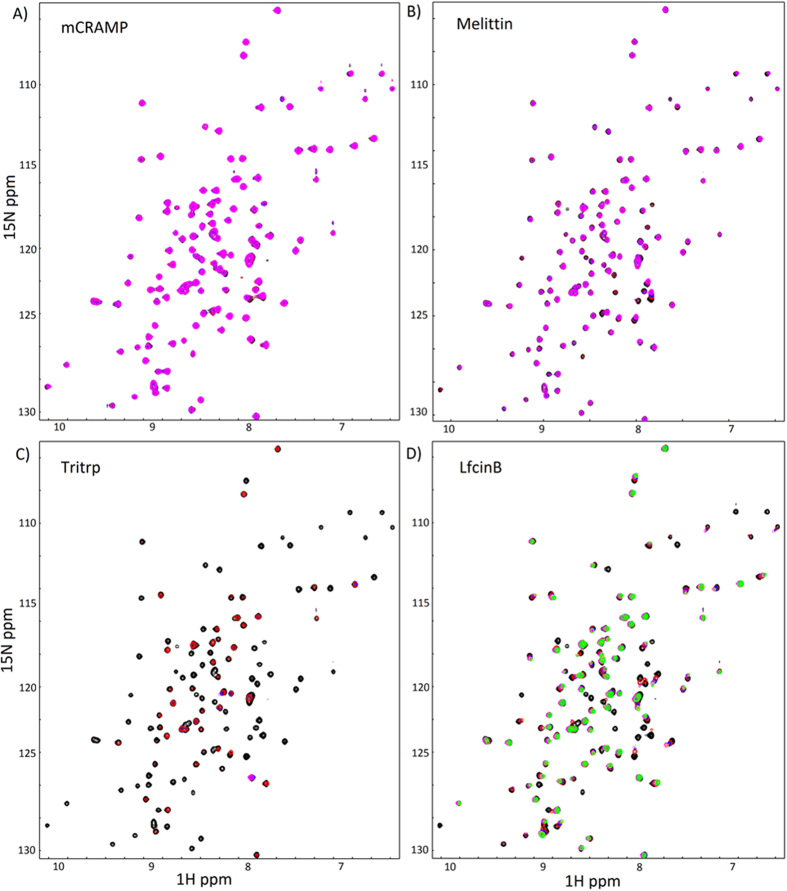
Overlays of HSQC spectra for ^15^N labelled SakΔN10 titrated with different antimicrobial peptides, acquired at 25 °C. (**A**) mCRAMP, (**B**) melittin, (**C**) Tritrp, and (**D**) LfcinB. Black peaks correspond to SakΔN10 in buffer alone. 0.5, 1.0, and 1.5 molar equivalent peptide added are shown in red, blue, and magenta, respectively. Additional titrations points were acquired for LfcinB with yellow and green peaks corresponding to 2.0 and 2.5 molar equivalents, respectively.

^1^H,^15^N HSQC spectra obtained for titrations of the peptide into ^15^N-labeled SakΔN10 samples are shown in Fig. 3. In these spectra, and with Sak to a lesser degree, some N-H peaks can be observed to undergo varied amounts of line broadening. This indicates that the interacting residues are in intermediate exchange on the NMR timescale. The magnitude of peak intensity losses for the affected residues is in general agreement with the different affinities for each peptide as determined from the ITC data. For mellitin and LfcinB, there are a few peaks that also undergo fast exchange between the unbound and bound conformations, allowing for the chemical shift perturbations to be followed.

**Figure 4:**
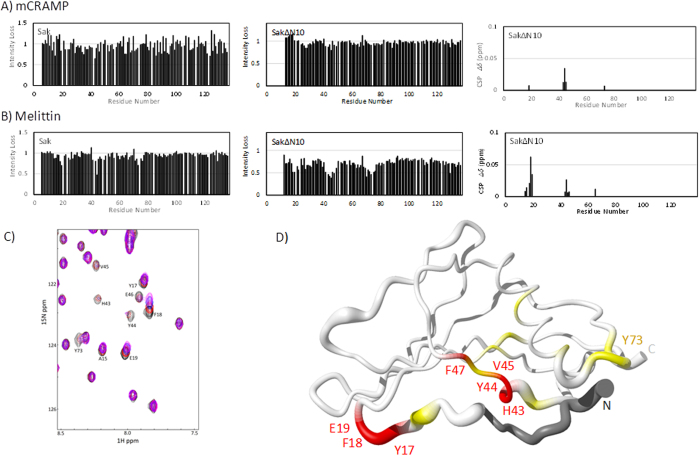
Peak intensity losses in the ^1^H,^15^N HSQC spectra of (**A**) mCRAMP and (**B**) melittin titrated into Sak (left panel) and SakΔN10 (middle panel) at 1.0 molar equivalent titration points. Corresponding chemical shift perturbations are shown for SakΔN10 (right panel). No such shifts occurred with Sak. (**C**) Selected region of the SakΔN10 HSQC spectra titrated with melittin with relevant peaks labeled. (**D**) Sausage diagram of Sak shaped according to the backbone distribution of the 20 lowest energy NMR structures (PDB ID 1SSN). Residues 1–10 are in dark grey, primarily affected residues identified in the mCRAMP and melittin titrations are labeled and coloured in red, less affected residues in yellow.

The spectra for full-length Sak remain mostly unchanged in titrations with the α-helical peptides mCRAMP and melittin, and there are no clear regions in the protein sequence with significant peak intensity loss (Fig. 4A,B). This result also occurs when mCRAMP is added to SakΔN10, however there is slight peak movement for Phe18, His43, Tyr44, Val45 and Tyr73. Residues 43 to 45 are part of a bulge region in staphylokinase (Leu40 to Glu46) of irregular secondary structure that follows β1 and precedes the short β2 strand (Phe47 to Ile49). The chemical shift perturbations seen here are very low in value compared to usual studies, which likely reflects the weak nature of these interactions.

In the titration of melittin to SakΔN10, His43 to Glu46 of the bulge region undergo both intermediate and fast exchange. There are more residues involved in the interaction than for mCRAMP. The peaks for Asp14 to Glu19 (excluding Ser16) from the N-terminal region are shifted during the titration, and there are additional peaks that undergo significant intermediate exchange. These new peaks, having lost >0.5 signal intensity at 1:1 melittin:SakΔN10, correspond to Ala70, Tyr73, and Lys74. Residues 69 to 76 form a loop region that joins the central α-helix to strand β3. Unsurprisingly, the residues that have increased solvent exposure after the deletion of residues 1–10 are 43 to 46 and 71 to 75 (Fig. 2C). These neighbouring segments in SakΔN10 can provide an interface for binding melittin, while mCRAMP seems to interact with residues 43 to 46 exclusively. In the bulge region, the side chains of Tyr44, Val45 and Phe47 offer sites for hydrophobic interactions, and Glu46 for electrostatic interactions with cationic AMPs. The low 70’s loop presents a less ideal area for AMP interaction because it contains a somewhat hydrophobic Tyr73 and the negative charge of Glu75 is offset by Lys74. In addition, there are peaks in residues corresponding to the end of the N-terminal fragment (Asp14 to Glu19) that are in intermediate and fast exchange. This region is well suited to extend the binding surface to accommodate a longer α-helical peptide such as melittin.

**Figure 5:**
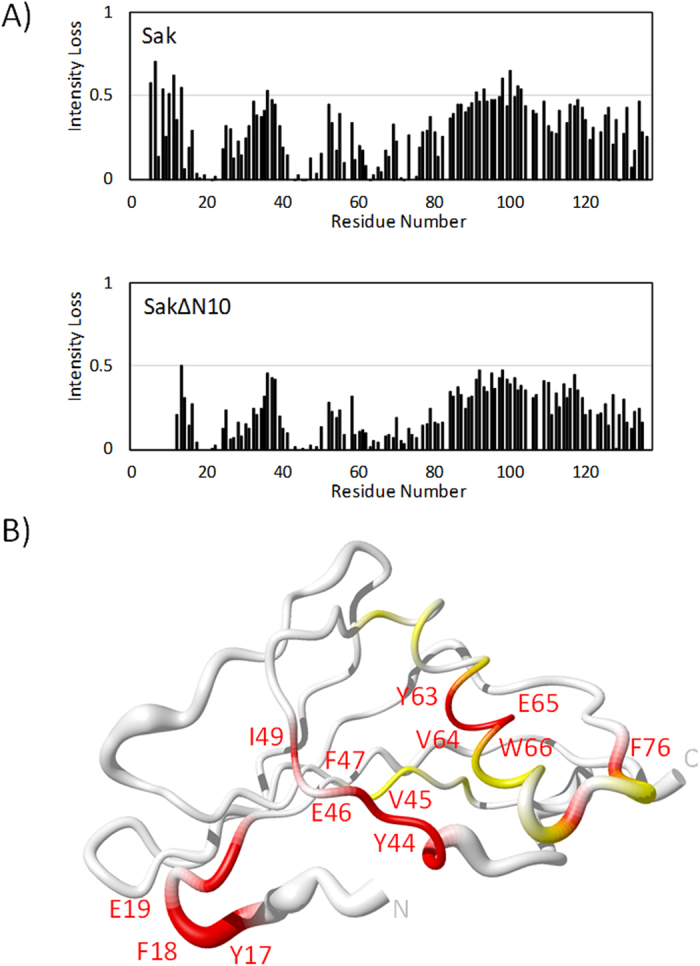
(**A**) Peak intensity losses in the ^1^H,^15^N HSQC spectra of Tritrp titrated into Sak (left panel) and SakΔN10 (right panel) at 0.5 molar equivalent titration points. (**B**) Backbone diagram of Sak (left) and SakΔN10 (right) highlighting affected residues in Tritrp interaction. Residues with normalized peak intensities less than 0.05 at 0.5 molar equivalent are in red; residues with normalized peak intensities between 0.05 and 0.1 are in yellow; residues 1–10 in Sak are in dark gray. Negatively charged and hydrophobic/aromatic residues at the interface are labeled for SakΔN10.

The HSQC peaks for Sak and SakΔN10 disappear very quickly upon addition of Tritrp such that the spectra are mostly blank after 1:1 Tritrp:SakΔN10 (Fig. 3C). Signal intensity losses at the 0.5 molar equivalence point are plotted and corresponding residues that have mostly disappeared, i.e. those with normalized peak intensities <0.1, are highlighted on the protein backbones in Fig. 5. Here, differences between Sak and SakΔN10 are not as prevalent as with melittin and mCRAMP. Hence, the potential binding surface for Tritrp is sufficiently large and accessible so that the first ten residues of Sak do not block this interaction, although the ITC data indicate that the binding is weakened. Interacting residues come from the segments that have been previously mentioned: the end of the N-terminal arm (Tyr17 to Glu19), the bulge region (Leu40 to Glu46), and the loop following the α-helix (Asp69 to Phe76). The interaction surface grows larger with residues from the α-helix, which contains the sole Trp residue of Sak at position 66. Altogether, this presents a continuous surface on Sak that has three negatively charged side chains, two long chain aliphatic side chains, and seven aromatic rings. This large hydrophobic area is poised to accommodate Tritrp, especially with opportunities for ring stacking interactions with the five aromatic side chains of the peptide.

**Figure 6:**
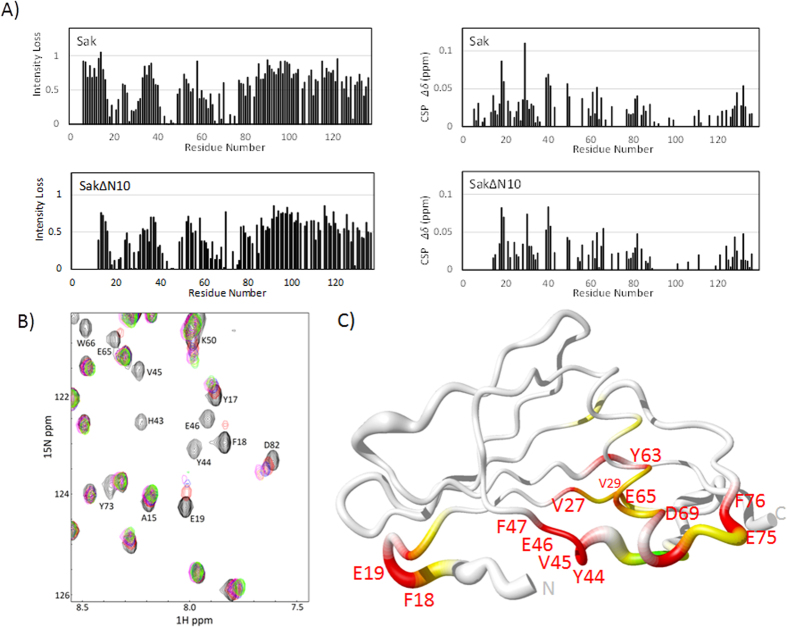
(**A**) Peak intensity losses (left panels) and chemical shift perturbations (right panels) in the ^1^H,^15^N HSQC spectra of LfcinB titrated into Sak (top panels) and SakΔN10 (bottom panels at 1.0 molar equivalent titration points. (**B**) Selected region of the SakΔN10 HSQC spectra titrated with LfcinB. (**C**) Backbone diagram of SakΔN10 highlighting important residues in LfcinB interaction. Residues with normalized peak intensities less than 0.15 at 1.0 molar equivalent are in red; residues with normalized peak intensities between 0.3 and 0.15 are in yellow; remaining residues with chemical shift perturbations greater than 0.05 are in green.

The interaction between LfcinB and staphylokinase illustrates another scenario for how the protein’s structure accommodates AMPs (Fig. 6). As seen in the HSQC overlay of the titration, both intermediate and fast exchange occur (Fig. 3D, plotted in Fig. 6A). At 2.5:1 peptide:protein, at least half of the peaks retain significant intensity, which is less drastic reduction than the signal disappearance observed with Tritrp. The mixture of LfcinB with SakΔN10 brings overall higher signal intensity losses compared to LfcinB with Sak, which reaffirms their difference in affinity, however the pattern of participating residues are mostly in agreement. A total of 75 out of the 115 assigned residues in SakΔN10 undergo some kind of chemical shift perturbations, which suggests that most of the protein may be experiencing some conformational rearrangement. Similar to Tritrp, the interfacial residues involve the bulge region (Leu40 to Phe47) and secondary sites are at the end of the N-terminal arm (Tyr17 to Glu19), the central α-helix (Tyr62 to Leu68) and the following loop that leads to strand β3 (Asp69 to Phe76). Additionally, the peaks for Val27, Val29 and Thr129 disappear almost completely at the first titration point. These residues belong to the adjacent anti-parallel strands β1 (V27, V29) and β5 (T129) and are buried in the interior of the protein, therefore it would be difficult for them to make direct interactions with LfcinB. Moreover, they serve as anchoring residues for the α-helix, therefore the loss in their signal intensities may be due to a movement or partial refolding of the α-helix as opposed to making direct contact with the peptide.

### Docking models for SakΔN10 complexes reveal two locations to accommodate AMPs

Using the experimental NMR titration data, we generated data-driven docking models for the complexes of SakΔN10 with three of the peptides studied here using structures available from the PDB and the HADDOCK web server^45^. The monomeric melittin template was taken from an X-ray crystal structure of the peptide in its aqueous tetrameric state^46^. It is mostly in an α-helical conformation, which is also expected for the peptide when it is membrane-bound. Tritrp requires a membrane-mimicking environment to fold into a converged conformation, and consequently the amphipathic micelle-bound structure of Tritrp^47^ was used as an approximation of the peptide in complex with SakΔN10. The amphipathic β-hairpin solution structure of LfcinB is conformationally restricted by a disulfide bond and was used in the docking calculations.

**Figure 7:**
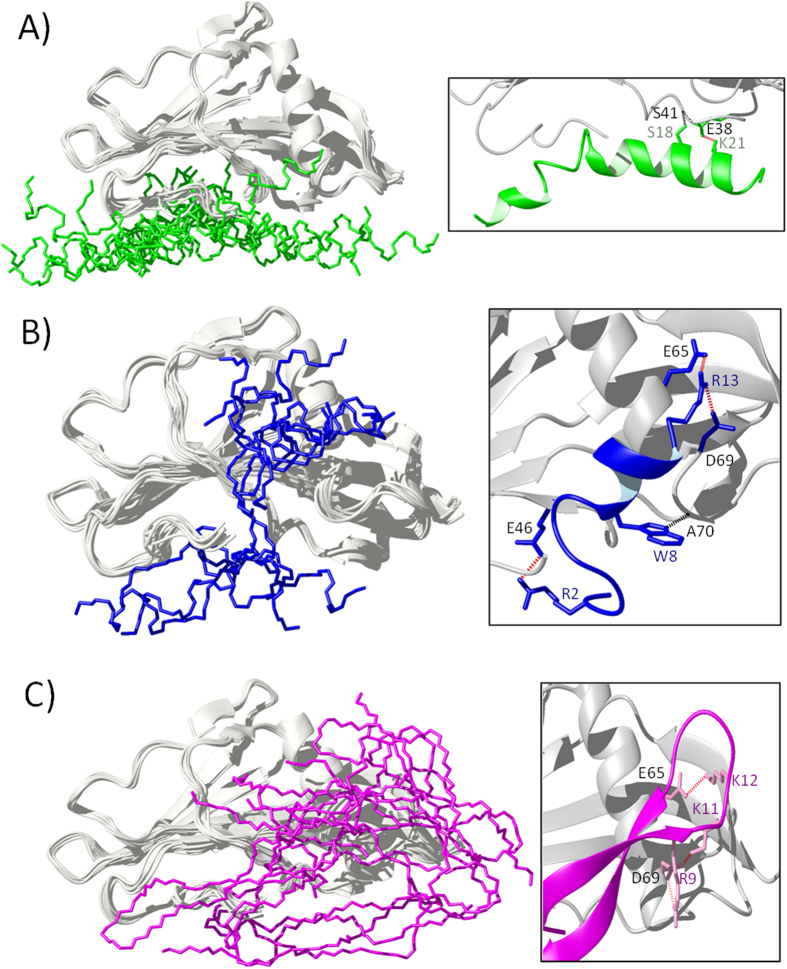
HADDOCK-calculated docking models of SakΔN10 with (A) melittin, (B) Tritrp, and (C) LfcinB. The left panels show the ten top scoring structure clusters superimposed on each other. The right panels show a close-up of the lowest energy complex model for each peptide with interacting side chains. Consistent intermolecular contacts within the structure clusters are indicated for hydrogen bonds (black dots) and ionic interactions (red dots).

The ten top scoring clusters of SakΔN10 in complex with each peptide are superimposed and shown in Fig. 7. Overall, SakΔN10 does not change conformation drastically with backbone root mean squared deviations less than 0.8 Å between the complexed and starting forms of the protein. In the models, melittin is spatially confined by interactions with the bulge region and the N-terminal arm of SakΔN10. Within the best scoring cluster of SakΔN1:melittin complexes, there is one consistent intermolecular electrostatic interaction between Lys21melittin and Glu38SakΔN10. The original structure of melittin consists of two α-helical segments connected by a slight hinge at Thr11-Gly12^46^. In the models with SakΔN10, the bending of the peptide about these residues becomes more pronounced. For Tritrp and LfcinB, SakΔN10 has several possible binding locations. However, the best scoring structure clusters of these complexes have the peptides resting on the α-helix of SakΔN10 and the subsequent loop, with at least three prominent electrostatic contacts from Glu65 and Asp69 to cationic residues from the peptides.

## Discussion

In the present study, our aim was to evaluate staphylokinase as a more general interacting partner for antimicrobial peptides of different structures and origin. Unexpectedly, the peptide that we had initially selected as a positive control, mCRAMP, did not show strong binding in our *in vitro* experiments. It is possible that additional factors may be involved in promoting its binding *in vivo*. During staphylococcal pneumonia in an intranasal infection mouse model, the expression of mCRAMP is upregulated and this in turn augments Sak-dependent plasminogen activation by an approximate two-fold factor^17^.

Regardless of the peptide, our data clearly show that SakΔN10 is a better binding partner compared to the full-length protein. The missing N-terminal portion of Sak has the sequence SSSFDKGKYK. With two aromatic rings and three cationic side chains, this sequence somewhat resembles that of an AMP itself and therefore this portion of the native protein could block a region on the protein that an incoming AMP would have to outcompete. The first fifteen residues exhibit high flexibility in the Sak structure as determined by X-ray crystallography and ^15^N NMR dynamics experiments^37^,^38^. However, the solution structure of Sak indicates that this section loosely folds back onto the protein with many observed NOEs from Phe4, Lys6, Lys8, Tyr9 and Lys10 to His43 and Tyr44^37^, two critical residues involved in AMP binding as identified by our NMR titration data. Although the cleavage at the Lys10-Lys11 peptide bond would normally occur during an activation step by plasmin while Sak is bound to it^1^,^48^, it seems that this N-terminal processing can be accomplished by a staphylococcal protease as well^42^. To date, very few proteins are known to interact directly with AMPs^49^,^50^ and to our knowledge, we have shown here for the first time the preferential binding of certain AMPs to a proteolytically processed form of an extracellular protein. It is interesting to speculate that this phenomenon may be more widespread, for example proteolysis of various chemokines is known to be quite common, which markedly changes their interaction properties^51^. Clearly future studies should consider the effects of proteolytic or other posttranslational modifications on the binding of AMPs to proteins in the circulatory system.

**Figure 8:**
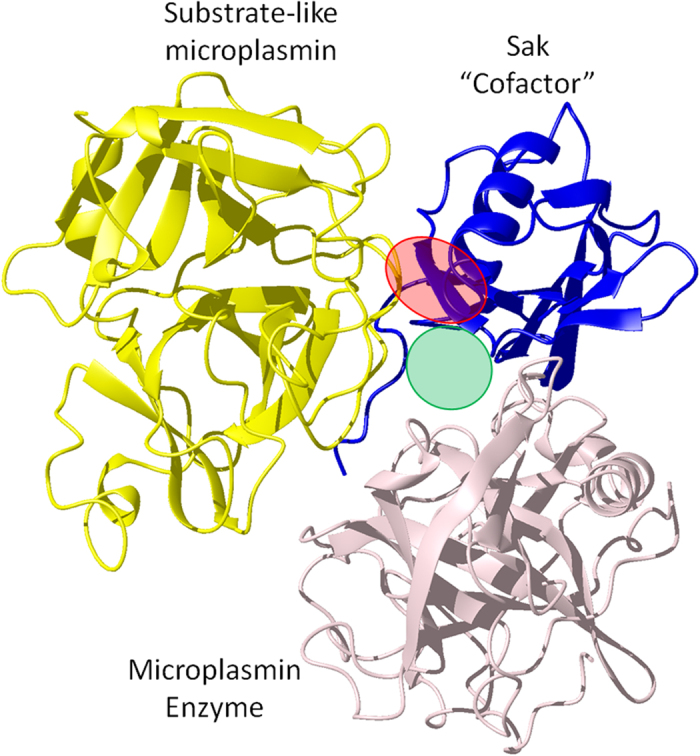
X-ray crystal structure of the ternary complex of the Sak/microplasmin activating enzyme unit bound to a microplasmin “substrate”. The high affinity site for AMPs, which overlaps with the activation epitope, is shown as a red circle on Sak. The lower affinity site for AMPs is shown as a green circle. PDB ID 1BUI^52^.

According to our findings, staphylokinase can interact with several AMPs using two distinct modes of binding. Tritrp and LfcinB are associated with a high affinity interaction type that includes several salt bridges with the α-helix of SakΔN10 (Fig. 7B,C). The interface area is large enough that the peptides are still able to bind to full Sak even though residues 1–10 of the N-terminal arm could give rise to some steric hindrance. Moreover, these two peptides occupy the same space and interact with the same region as a plasminogen substrate during enzymatic action by the plasmin/Sak complex (Fig. 8)^52^. In this complex, the active site belongs to plasmin while Sak acts as a cofactor that restricts a binding subsite for the incoming substrate. According to an X-ray structure of the ternary complex, where smaller microplasmin proteins were used as substitutes for plasmin (PDB ID 1BUI)^52^, the plasminogen side chains His569, Pro570 and His571 extend into the hydrophobic groove consisting of Tyr62, Trp66, Ala70 and Tyr73 from the Sak α-helix-β3 loop. Furthermore, mutational studies have identified residues in this so-called “activation epitope” that affect plasminogen activation without changing the affinity of the plasmin/Sak enzyme complex: Val45, Phe47, Tyr62, Tyr63, Trp66, and Ala70. Our experimental data also shows that interactions between LfcinB and the α-helix can affect the conformation of residues belonging to the β-sheet on the opposite face of the protein. The strong binding of LfcinB and Tritrp to the activation epitope is likely to inhibit enzymatic activity in the same manner as the binding of HNP-1^15^.

In the case of melittin and mCRAMP, there is weaker affinity with more pronounced inhibition by the N-terminal segment in full Sak. These peptides belong to the canonical class of AMPs that gain α-helical structure in a membranous environment, however it is unknown if they fold into such conformations upon binding to SakΔN10. The attachment location for these peptides is more restricted to the opposite face of the bulge region away from the activation epitope and has a limited interface composed of residues in the bulge area and Tyr17-Glu19 of the flexible N-terminal arm (Fig. 7A). This positions the peptide in proximity with the plasmin partner that attaches to Sak that forms the activating complex, however this may not present a direct interference in binding as plasmin has several contacts with the larger face of the β-sheet, specifically with strands β3, β5, β1 and β2^52^. In the plasmin/Sak enzyme complex, there are salt bridges from Lys769 and Arg719 of plasmin to Glu19 and Glu46 of Sak, respectively. The effect of plasmin binding causes residues 43 and 44 of Sak to protrude into the activation epitope and restricts the substrate binding pocket. Furthermore, this smaller space more closely resembles the binding pocket offered by urokinase and tissue plasminogen activator which are the native plasminogen activators in humans. Given the slight effects that mCRAMP and melittin have on His43 and Tyr44 of SakΔN10 without influencing many more residues on the face of the activation epitope, we hypothesize that the peptides facilitate the plasmin-mediated fine-tuning of the substrate pocket. This would explain the doubled plasminogen activating activity of Sak in the presence of mCRAMP that promotes *S. aureus* invasion^17^.

The bulge residues 43 to 47 remain the focal point for both types of interaction with Sak, and the removal of the N-terminal arm from these residues facilitates either substrate or AMP binding. Previous molecular models have emphasized the importance of these residues, with His43 and Tyr44 making crucial cation-pi and pi-pi interactions with the Trp215 side chain of plasmin^2^. However, we did not observe any of the Trp indole rings of Tritrp or LfcinB to be oriented in such a manner in our data-driven docking models.

The structural analysis of staphylokinase presented here reveals versatility in its intermolecular interactions, and the two different modes of binding for AMPs may lead to different consequences for substrate binding and functional activation. The clinical development of Sak as a thrombolytic agent may be aided by an accompanying peptide that boosts its activity such as mCRAMP or melittin, however the latter is a strongly cytotoxic peptide, limiting its clinical usefulness. As well, we should re-emphasize the role of staphylokinase as an AMP sequestering agent. We observed a 1:1 binding ratio for the peptides studied here, however this may not be the case for all peptides. In fact, a molar ratio as low as 1:6 Sak:HNP was found to neutralize the peptide’s bactericidal activity, suggesting that Sak can influence oligomerization of some AMPs^15^.

Altogether, our results reveal two major binding pockets that are present on the surface of staphylokinase to accommodate interactions with antimicrobial peptides, and large anionic surfaces on the protein allow for the binding of peptides of different sizes and structures. These results highlight the importance of SakΔN10 as an important part of the resistance mechanism of *S. aureus* against host innate immune defenses.

## Materials and Methods

Most peptides were purchased from Genscript (Piscataway, NJ) as custom-synthesized peptides synthesized using standard 9-fluorenylmethyoxycarbonyl (Fmoc) chemistry and they were purified by HPLC to >95% purity. Melittin from honey bee venom (>85% purity) was purchased from Sigma-Aldrich. The amino acid sequences of the peptides are as follows: LfcinB, FKCRRWQWRMKKLGAPSITCVRRAF with a disulfide bond between the underlined Cys residues; mCRAMP, GLLRKGGEKIGEKLKKIGQKIKNFFQKLVPQPEQ; melittin, GIGAVLKVLTTGLPALISWIKRKRQQ-NH_2_; Tritrp, VRRFPWWWPFLRR-NH_2_. For the purpose of this study, Tritrp refers to the C-terminally amidated version of tritrpticin which has more potent antimicrobial properties than the original non-amidated peptide^47^,^53^. The concentrations for most of the peptides were determined by UV absorption at 280 nm using molar extinction coefficients determined by ProtParam^54^. In the case of mCRAMP which does not contain any Trp or Tyr residues, UV absorption at 214 nm was measured and a molar extinction coefficient was calculated from its amino acid content^55^.

### Protein expression and purification

A synthetic gene for staphylokinase with optimized codons for expression in *Escherichia coli* was purchased from GeneArt (Life Technologies). Staphylokinase genes have been cloned and sequenced from different origins: the bacteriophages *sakφ*C and *sak*42D, and the genomic DNA of a lysogenic *S. aureus* strain (sakSTAR)^56^. These indicate amino acid sequence differences at three positions in the coding sequences. In this study, we have worked with the sakSTAR protein sequence as it is the most commonly used variant^56^. The gene was subcloned into a pET29b (Novagen), giving the expressed protein a C-terminal His_6_-tag when expressed in *E. coli* BL21 (DE3) cells. Site-directed mutagenesis was performed with designed primers to make a gene construct for SakΔN10, the more active form of Sak with its first ten residues deleted.

^15^N-labeled or ^13^C,^15^N-labeled Sak or SakΔN10 were expressed in a minimal M9 medium containing 0.5 g/L ^15^NH_4_Cl, and 3 g/L of either ^13^C-labeled or unlabeled glucose. At an optical density at 600 nm of ~0.6, the cells were induced with 0.5 mM isopropyl β-D-1-thiogalactopyranoside for 4 h at 37 °C. The cells were collected by centrifugation and put through at least 3 passes through a French press in lysis buffer: 20 mM Tris, 100 mM NaCl, pH 7.4. The cell lysate was then clarified by high speed centrifugation, after which the supernatant was applied onto a column with chelating-Sepharose fast flow resin (GE Healthcare) loaded with NiCl_2_. The column was washed with a buffer containing 50 mM imidazole, and the protein of interest was eluted with 400 mM imidazole. The purified protein was dialyzed extensively in 8 mM NH_4_HCO_3_ and lyophilized. SDS-PAGE indicated that the protein was >95% pure.

### ITC

All ITC runs were performed at 25 °C on a MicroCal VP-ITC microcalorimeter (Malvern, Westborough, MA). 200–400 μM peptide was titrated into 8 μM SakΔN10 or Sak in 20 mM HEPES, pH 7.4. In preliminary trials, the inclusion of salt (100 mM NaCl) in the ITC buffer was found to promote peptide aggregation in the injection syringe as indicated by endothermic heats of dilution in peptide-to-buffer control experiments. Data analysis was performed using the MicroCal Origin software using the “One Set of Sites” binding model. At least two experiments were performed for each titration. Statistical analysis was performed to confirm the statistical validity of the differences in the K_D_ values determined for LfcinB and Tritrp binding to SakΔN10 or Sak. p-values were calculated from Student’s *t-tests* at the 95% confidence level with a one-tailed hypothesis.

### NMR measurements

All NMR experiments were performed at 298 K on a Bruker Avance 700 MHz spectrometer equipped with a triple resonance inverse Cryoprobe with a single axis z-gradient. An initial 0.15–0.20 mM ^13^C,^15^N-labeled Sak sample was dissolved in 20 mM sodium phosphate, 100 mM NaCl, 1 mM EDTA, and 0.5 mM 2,2-dimethyl-2-silapentane-5-sulfonate (DSS), pH 6.9, supplemented with 10% ^2^H_2_O. To complete the assignment of the main chain NMR signals, a two-dimensional [^1^H-,^15^N]-HSQC and three-dimensional HN(CA)CO, HNCO, HNCA, HN(CO)CA, HNCACB, and CBCA(CO)NH experiments were acquired with a non-uniform sampling scheme in the indirect dimensions. All spectra were processed using NMRPipe^57^ and analyzed using the NMRView software package^58^. The three-dimensional experiments with non-uniform sampling were reconstructed with MDDNMR^59^ interfaced with MDDGUI^60^. The backbone assignments for Sak were compared and verified with those reported in a previous publication^37^.

In peptide titration experiments, [^1^H-,^15^N]-HSQC NMR spectra were acquired after aliquots of concentrated peptide samples were titrated in 0.5 molar equivalents to the Sak NMR samples. After each titration point, the pH was monitored and kept at 6.9. Chemical shift perturbations (CSPs, *Δδ*) were calculated as a weighted average chemical shift difference of ^1^H and ^15^N resonances between unbound Sak and peptide-bound Sak^61^, using equation 1:


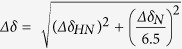


### Data-driven HADDOCK modeling

Docking models for SakΔN10 in complex with melittin, Tritrp and LfcinB were generated using the HADDOCK 2.2 (High Ambiguity Driven protein-protein DOCKing) web server^45^. The SakΔN10 structure was modified from the mean solution structure of Sak (PDB ID 1SSN)^37^ with residues 1–10 artificially deleted. The sources of the monomeric peptide structures used in our calculations are: the X-ray crystal structure of melittin in its aqueous tetrameric form (PDB ID 2MLT)^46^, the micelle-bound NMR structure of Tritrp (PDB ID 2I1D)^47^, and the aqueous NMR solution structure of LfcinB (PDB ID 1LFC)^35^. The participating residues of SakΔN10 were selected from the NMR titration data for each peptide. For SakΔN10:melittin, the active residues were defined to have <0.6 normalized peak intensities at 0.5:1 peptide to protein: Y17, F18, E19, H43, Y44, E46, V45, and K74. The passive residues were new residues with <0.6 normalized peak intensities at 1:1 peptide to protein and/or underwent significant chemical shift perturbation: D14, A15, G22, V27, S41, F47, A70, Y73, E75, and F76. For SakΔN10:Tritrp, active residues have <0.05 normalized peak intensities at 0.5:1 peptide to protein: Y17, F18, E19, T21, G22, H43, Y44, V45, E46, F47, I49, Y63, V64, E65, W66, A72, and F76. Passive residues had normalized peak intensities between 0.05 and 0.1 at the same titration point: M26, V27, V29, T56, K59, Y62, A67, L68, T71, K74, and E75. Please note that the selection criteria of passive residues here differs from melittin and LfcinB due to the overabundance of residue peaks with drastic intensity losses at 1:1 peptide to protein. For SakΔN10:LfcinB, active residues have <0.1 normalized peak intensities at 0.5:1 peptide to protein: V27, V29, Y44, V45, E46, A67, T71, A72, and K74. Passive residues were new residues with <0.1 normalized peak intensities at 1:1 peptide to protein and/or underwent significant chemical shift perturbation: F18, E19, T30, L39, L40, S41, F47, W66, D69, and E74. Due to the absence of experimental information regarding the interacting residues from the peptides, they were all considered to be equally active in the HADDOCK calculations.

## Additional Information

**How to cite this article**: Nguyen, L. T. and Vogel, H. J. Staphylokinase has distinct modes of interaction with antimicrobial peptides, modulating its plasminogen-activation properties. *Sci. Rep.* **6**, 31817; doi: 10.1038/srep31817 (2016).

## Supplementary Material

Supplementary Information

## Figures and Tables

**Table 1:** Dissociation constants, stoichiometries and thermodynamic parameters determined for antimicrobial peptide binding to staphylokinase as determined by isothermal titration calorimetry.

Sample + titrant	*K*_*D*_ (μM)	*N*	*ΔH* (cal mol^−1^)	*ΔS* (cal mol^−1^)
Sak + LfcinB	27.3	0.98	−4.68 × 10^3^	5.18
Sak + Tritrp	11.2	1.16	−3.02 × 10^3^	12.5
Sak + mCRAMP	no interaction			
Sak + melittin	weak interaction			
SakΔN10 + LfcinB	0.37*	0.99	−3.01 × 10^4^	19.3
SakΔN10 + Tritrp	2.42*	1.14	−7.01 × 10^3^	2.19
SakΔN10 + mCRAMP	weak interaction			
SakΔN10 + melittin	weak interaction			

*p-values < 0.05 against corresponding *K*_*D*_’s with Sak using the *t-test* at the 95% confidence level.
