# Supplementary material for: Staphylokinase has distinct modes of interaction with antimicrobial peptides, modulating its plasminogen-activation properties
Source: Sci Rep. 2016 Aug 24;6:31817. doi: 10.1038/srep31817 (PMC4995489; doi:10.1038/srep31817)
Supplement: Supplementary Information [file srep31817-s1.doc]

**Staphylokinase has distinct modes of interaction with antimicrobial peptides, modulating its plasminogen-activation properties**

Leonard T. Nguyen and Hans J. Vogel

Supplementary Figure 1. Assigned ^15^N-^1^H HSQC spectra of A) Sak and B) SakΔN10.

Supplementary Figure 1A

Supplementary Figure 1B

Supplementary Figure 1. Assigned ^15^N-^1^H HSQC spectra of A) Sak and B) SakΔN10.
